# Adolescent experiences of mistreatment during childbirth in health facilities: secondary analysis of a community-based survey in four countries

**DOI:** 10.1136/bmjgh-2021-007954

**Published:** 2022-03-21

**Authors:** Theresa Azonima Irinyenikan, Adeniyi Kolade Aderoba, Olufunmilayo Fawole, Olusoji Adeyanju, Hedieh Mehrtash, Kwame Adu-Bonsaffoh, Thae Maung Maung, Mamadou Dioulde Balde, Joshua P Vogel, Marina Plesons, Venkatraman Chandra-Mouli, Özge Tunçalp, Meghan A Bohren

**Affiliations:** 1 Department of Obstetrics and Gynaecology, Faculty of Clinical Sciences, University of Medical Sciences, Ondo City, Ondo State, Nigeria; 2 Department of Obstetrics and Gynaecology, University of Medical Sciences Teaching Hospital Complex, Akure, Ondo State, Nigeria; 3 Department of Obstetrics and Gynaecology, Mother and Child Hospital, Akure, Ondo State, Nigeria; 4 National Perinatal Epidemiology Unit, Nuffield Department of Population Health, University of Oxford, Oxford, UK; 5 Department of Epidemiology and Medical Statistics, Faculty of Public Health, College of Medicine, University of Ibadan, Ibadan, Nigeria; 6 Adeoyo Maternity Teaching Hospital, Ibadan, Nigeria; 7 Department of Sexual and Reproductive Health and Research, including UNDP/UNFPA/UNICEF/WHO/World Bank Special Programme of Research, Development and Research Training in Human Reproduction (HRP), World Health Organization, Geneva, Switzerland; 8 Department of Obstetrics and Gynecology, University of Ghana Medical School, Accra, Ghana; 9 Department of Obstetrics and Gynecology, Korle-Bu Teaching Hospital, Accra, Ghana; 10 Department of Medical Research, Yangon, Myanmar; 11 Cellulle de Recherche en Sante de la Reproduction en Guinee (CERREGUI), Conakry, Guinea; 12 Maternal, Child and Adolescent Health Program, Burnet Institute, Melbourne, Victoria, Australia; 13 Gender and Women's Health Unit, Centre for Health Equity, Melbourne School of Population and Global Health, University of Melbourne, Melbourne, Victoria, Australia

**Keywords:** maternal health, community-based survey, epidemiology, health services research, public health

## Abstract

**Introduction:**

Pregnancy and childbearing among adolescents—especially younger adolescents—is associated with health complications and lost opportunities for education and personal development. In addition to established challenges adolescents and young women face in sexual and reproductive healthcare, evidence suggests that they also face mistreatment during childbirth.

**Methods:**

This is a secondary analysis of the WHO study ‘How women are treated during facility-based childbirth’ cross-sectional community survey in Ghana, Guinea, Myanmar and Nigeria. We used descriptive analysis to assess experiences of mistreatment among adolescents (15–19 years) and young women (20–24 years) and multivariable logistic regression models to assess the association between experiences of mistreatment and satisfaction with care during childbirth.

**Results:**

862 participants are included (15–19 years: 287, 33.3%; 20–24 years: 575, 66.7%). The most common mistreatment was verbal abuse (15–19 years: 104/287, 36.2%; 20–24 years: 181/575, 31.5%). There were high levels of poor communication (15–19 years: 92/287, 32.1%; 20–24 years: 171/575, 29.7%), lack of supportive care (15–19 years: 22/287, 42.5%; 20–24 years: 195/575, 33.9%) and lack of privacy (15–19 years: 180/287, 62.7%; 20–24 years: 395/575, 68.7%). Women who were verbally abused were less likely to report satisfaction with care (adjusted OR (AOR): 0.19, 95% CI: 0.12 to 0.31) and less likely to recommend the facility (AOR: 0.24, 95% CI: 0.15 to 0.38). There were similar reports among those who were physically abused, had long waiting time, did not mobilise and did not give consent for vaginal examinations.

**Conclusion:**

Our study shows that adolescents and young women mistreatment during childbirth, contributing to low satisfaction with care. It is critical to recognise adolescents and young women’s unique needs in maternal healthcare and how their needs may intersect with social stigma around sex and pregnancy.

**Key questions**

**What is already known?**

- Despite considerable progress in preventing adolescent pregnancy over the past 25 years, an estimated 12 million girls aged 15–19 years give birth each year.
- Adolescents and young women are less likely than adult women to get the maternal healthcare and support they need in health facilities.
- Adult women who experience mistreatment during childbirth are more likely to report low satisfaction with care, but the extent and type of adolescent experiences of mistreatment have not been explored.

**What are the new findings?**

- This study explored the experiences of mistreatment during childbirth among adolescents (15–19 years) and young women (20–24 years), and their satisfaction with care, using community-based cross-sectional surveys in Ghana, Guinea, Myanmar and Nigeria.
- We found that adolescent experiences of mistreatment were common, particularly verbal abuse, poor communication, lack of supportive care and lack of privacy.
- Adolescents and young women who reported experiences of mistreatment—especially the better educated young women—were more likely to report lower satisfaction with the care they received.

**Key questions**

**What do the new findings imply?**

- While adolescents and young women should be supported to use their own agency in maternal healthcare seeking, it is critical for health workers and other stakeholders to recognise adolescents’ unique needs and how their needs may intersect with social stigma around sex and pregnancy in adolescence.
- More work is needed to ensure that adolescent and young women’s needs and preferences are met in maternity care settings globally, particularly around improving equitable and non-discriminatory care.

## Introduction

Across low- and middle-income countries, about 12 million adolescents aged 15–19 years give birth each year and at least 10 million unintended pregnancies occur each year in the same age group.^1^ Currently, maternal mortality remains one of the leading global causes of death among adolescents, especially in sub-Saharan Africa, and is considered an urgent public health challenge.^1 2^ There are several factors contributing to adolescent pregnancy that vary between and within countries and communities, including the pressure of early marriage and early childbearing,^3–6^ and limited prospects for education or employment that make early marriage or childbearing a preferred option.^7^ According to the most recent estimates, the percentage of women aged 20–24 who gave birth before age 18 years is 5% in Myanmar, 17% in Ghana, 29% in Nigeria and 40% in Guinea.^8^

Adolescents and young women who are pregnant require special attention due to additional needs around social support, and increased risks of serious severe and lasting health and social problems for both themselves and their babies.^9^ Compared with adult women, adolescents and young women have higher risks of experiencing maternal complications such as eclampsia, obstructed labour and puerperal sepsis, as well as newborn complications such as low birth weight, preterm birth and severe neonatal complications.^9^ Disappointingly, their particular needs have largely been neglected and as a result, they are less likely than adult women to get the maternal healthcare and support they need from health services during pregnancy and childbirth.^2 10^ The lasting social and economic consequences for pregnant adolescents, and especially unmarried pregnant adolescents are profound. For example, adolescent pregnancy often leads to discontinuation of schooling, thus jeopardising future education and employment,^11^ and girls pregnant before 18 years are more likely to experience intimate partner violence.^12^

The mistreatment of women during childbirth in health facilities has received increasing attention in recent years. A mixed-methods systematic review by Bohren and colleagues developed a typology of what constitutes mistreatment, including physical abuse, verbal abuse, stigma and discrimination, failure to meet professional standards of care, poor rapport between women and providers, and health system conditions and constraints.^13^ To address these challenges, WHO issued a statement in 2014 on the prevention and elimination of mistreatment during childbirth,^14^ as well as the 2018 recommendations on intrapartum care for a positive pregnancy experience, which specifically includes respectful maternity care.^15^ Recent evidence from a WHO multicountry study showed that over one-third of women of reproductive age (15–49 years) reported experiencing mistreatment during childbirth across four countries, Ghana, Guinea, Myanmar and Nigeria.^16^ This study found that compared with older women (≥30 years), adolescents (15–19 years) were almost twice as likely to experience physical abuse, and adolescents with no education were over three times more likely to experience verbal abuse.^16^ The reasons for poorer treatment of adolescents and young women during childbirth may be related to sociocultural and provider prejudices about their engagement in premarital or early sex and are compounded by lower educational attainment, lower socioeconomic status and limited autonomy.^17 18^ Mistreatment of adolescents and young women during childbirth is thus doubly problematic, as it compounds existing health and social burdens faced by this age group.^19^ This poor quality care can have lasting detrimental impacts on future health-seeking behaviour and trust in the health system.

To address existing gaps in knowledge and provide information for future policy and programming on adolescents’ maternal health, this analysis aims to describe adolescent experiences of mistreatment during childbirth, their satisfaction with care, and explore factors that contribute to these experiences.

## Methods

### Study design and setting

This is a secondary analysis of the cross-sectional, community-based survey component of the WHO multicountry study ‘How women are treated during facility-based childbirth,’ which was designed to develop and validate two tools (labour observation and community-based survey) to measure the mistreatment of women during childbirth in health facilities in four countries (Ghana, Guinea, Myanmar and Nigeria).^16^ In advance of the measurement study, we conducted a formative phase consisting of systematic reviews^13 20^ and primary qualitative research^17 21–26^ to understand the context and develop the measurement tools.^27^ An author reflexivity statement reflecting on the dynamics of our international partnership is available in online supplemental appendix 1.

10.1136/bmjgh-2021-007954.supp1 Supplementary data

This analysis focused on the cross-sectional, community-based survey data only, and the detailed methodology has been published elsewhere.^16 27^ Data collection took place in Nigeria from 19 September 2016 to 26 February 2017, in Ghana from 1 August 2017 to 18 January 2018, in Guinea from 1 July to 30 October 2017 and in Myanmar from 26 June to 5 September 2017. The study sites were located in urban areas and were both secondary and tertiary facilities with more than 200 births per month. Some facilities provided privacy with the use of curtains while some did not have curtains nor partitioning to allow for privacy. The selected facilities had well-defined catchment areas with a population of over a million and facility-based childbirth in the catchment areas ranged from 35% to 90% across countries.^16^

### Participants and sample size

Women were eligible for enrolment in the study if they were admitted for childbirth in a study health facility, were ≥15 years, provided written informed consent and able to participate and for those who could not read the consent form and sign, the contents of the consent form was read out to them and their relative and they were asked to thumb print to give their consent. Women were also eligible if they resided in the predefined facility catchment area after birth (defined for each health facility) and provided sufficient contact information for follow-up after discharge. Women were not eligible if they were admitted for reasons other than childbirth, were a first-degree relative of a facility employee (eg, mother, sister, daughter, cousin), were distressed or otherwise unable to reasonably provide consent or lived outside the predefined catchment area for that health facility. The sample size for the community survey was 2672, out of which 862 were adolescents or young women (n=287 aged 15–19 years and n=575 aged 20–24 years), and included in this analysis.

### Study procedures

All women admitted to the health facility during the study period were assessed for eligibility. Those who met the eligibility criteria and agreed to participate were enrolled, and contact information was obtained. For the community survey, women were contacted to schedule a time at up to 8 weeks postpartum for a face-to-face individual interview. The women were contacted through the telephone numbers they provided before leaving the hospital and those who could not be reached through their telephone numbers were traced to their homes through the addresses they provided before leaving the hospital. Interviewers were trained female researchers who had a social science or public health background and were neither clinicians nor health workers. The choice of interviewers was to encourage women to speak more freely and openly about their care experience (eg, reduce the risk of social desirability bias). Recruitment continued until the planned per-facility sample size was reached.

### Measurement, management and analysis

The community survey tool is publicly available and captures information on a woman’s sociodemographic information, obstetric history, birth experiences (including mistreatment, vaginal examinations, companionship and pain relief), childbirth outcomes, childbirth interventions, postpartum depression, future childbearing intentions and satisfaction with care.^27^ Data were collected using digital, tablet-based tools (BLU StudioXL 2, Android OS; BLU Products, Miami, Florida, USA) and using OpenClinica open source software for data collection and management (Open Clinica, Waltham, Massachusetts, USA). Data were prospectively submitted to a WHO central server using a 3G-cellular connection or wireless internet.

We used descriptive analysis to assess the sociodemographic and obstetric characteristics of adolescents and young women by country for ages 15–24 years, and to explore adolescent experiences of mistreatment by country, disaggregated in 15–19 years and 20–24 years age groups. We used multivariable logistic regression models to assess adolescents’ and young women’s overall satisfaction with the care thy received during childbirth. We chose an indicator from each domain of mistreatment to assess where there was sufficient sample (n>200 women) to assess the association with satisfaction with care. The outcome was using the 5-point Likert satisfaction of care scale: strongly agreed/agreed (referred to as ‘satisfied with care’) and strongly disagreed/disagreed/neutral (‘neutral or not satisfied with care’), while adjusting for marital status, education, previous birth and country. The neutral response option was grouped with ‘no’ as it has been shown in previous respectful maternity care cognitive testing that women may not express their concerns. Therefore, it would be best to dichotomise Likert scales to better understand responses across different contexts.^28^

Similarly, logistic regression was conducted to assess adolescents and young women’s recommendation of the health facility to others using the following outcomes: strongly agreed/agreed (‘will recommend the facility to others’) and strongly disagreed/disagreed/neutral (‘neutral or will not recommend the facility to others’), while adjusting for marital status, education, previous births and country. Similar to our previous analysis in this project,^29^ marital status, education, previous births and country were specified as independent variables based on the results of this project’s primary study.^16^ Furthermore, we aimed to explore if the dose-response relationship between these variables was amplified for this population of adolescent and young women, as compared with the larger population of women where findings have been published.^29^ Both models did not adjust for age due to homogeneity in the sample, and because adolescents and young women have been previously explored compared with the older women in the larger study.^16^ All data were analysed using Statistical Package for Social Sciences (SPSS) Windows 23 and SAS 9.4.

### Patient and public involvement

A technical consultation with representatives from advocacy groups, non-governmental organisations, research organisations, universities, professional associations and United Nations agencies was held at WHO in November 2013 and informed the design of this study. Women who recently gave birth were involved in content validity testing and providing feedback on the validity testing of the community survey tool.

## Results

We included all 862 adolescents and young women aged 15–24 years in this analysis, representing 32.3% of the original sample of 2672 women. Table 1 shows the sociodemographic characteristics of participants by country. Of the 862 participants, there were 287 (33.3%) between the ages of 15–19 years and 575 (66.7%) between the ages of 20 and 24 years. Most participants were married (n=723/862, 83.9%), except in Ghana where a comparatively smaller proportion of participants were married (n=123/208, 59.1%). Most participants had some or complete secondary education (n=548/862, 63.6%) across countries, except for Guinea where most had only primary or no education (n=190/359, 53.0%). Most participants had only one previous birth (n=703/862, 81.6%) across countries, with 18.4% (n=159/862) having two or more previous births. Most participants had a vaginal birth for the most recent birth (n=730/862, 84.7%).

**Table 1:** Sociodemographic characteristics and obstetric history of adolescents aged 15–24 years, by country

	Ghana, n (%), N=208	Guinea, n (%), N=359	Nigeria, n (%), N=89	Myanmar, n (%), N=206	Total n (%), N=862
Age					
15–19 years	60 (28.8)	173 (48.2)	15 (16.9)	39 (18.9)	287 (33.3)
20–24 years	148 (71.2)	186 (51.8)	74 (83.1)	167 (81.1)	575 (66.7)
Marital status					
Single	84 (40.4)	33 (9.2)	16 (18%)	5 (2.4%)	138 (16.0%)
Married	123 (59.1)	326 (90.8)	73 (82)	201 (97.6)	723 (83.9)
Missing/unknown	1 (0.5)	0 (0)	0 (0)	0 (0)	1 (0.1)
Educational status					
No education	4 (1.9)	118 (32.9)	0 (0)	3 (1.5)	125 (14.5)
Some primary education	16 (7.7)	72 (20.1)	2 (2.2)	28 (13.6)	118 (13.7)
Some secondary education	100 (48.1)	114 (31.8)	14 (15.7)	60 (29.1)	288 (33.4)
Complete secondary education	71 (34.1)	40 (11.1)	60 (67.5)	89 (43.2)	260 (30.2)
Complete tertiary education	15 (7.2)	9 (2.5)	13 (14.6)	26 (12.6)	63 (7.3)
Vocational/unknown	2 (1.0)	6 (1.7)	0 (0)	0 (0)	8 (0.9)
Previous number of births					
1	183 (88.0)	273 (76.0)	76 (85.4)	171 (83.0)	703 (81.6)
2	18 (8.7)	52 (14.5)	11 (12.4)	28 (13.6)	109{12.6)
3	6 (2.9)	24 (6.7)	2 (2.2)	5 (2.4)	37 (4.3)
≥4	1 (0.5)	10 (2.8)	0 (0)	2 (1.0)	13 (1.5)
Mode of birth					
Vaginal birth	184 (88.5)	315 (87.7)	83 (93.3)	148 (71.8)	730 (84.7)
Caesarean section	24 (11.5)	43 (12.0)	6 (6.7)	58 (28.2)	131 (15.2)
Missing/unknown	0 (0)	1 (0.3)	0 (0)	0 (0)	1 (0.1)
No of babies*†					
1 (singleton)	205 (98.6)	353 (98.3)	88 (98.%)	203 (98.5)	849 (98.5)
2 (twins)	3 (1.4)	4 (1.7)	1 (1.1)	3 (1.5)	11 (1.5)
Sex of baby*†					
Female	99 (46.9)	161 (4.6)	47 (52.2)	98 (46.8)	401 (46.0)
Male	11 (52.6)	198 (4.8)	43 (47.8)	111 (53.1)	467 (53.6)
Unknown	1 (0.5)	2 (0.6)	0 (0)	0 (0)	3 (0.3)
Timing of breastfeeding initiation*					
Within 1 hour	91 (43.8)	138 (38.4)	30 (33.7)	145 (70.4)	404 (46.9)
Between 1 and 24 hours	87 (41.8)	187 (52.1)	46 (51.7)	41 (19.9)	361 (41.9)
Between 24 hours and 1 week	24 (11.5)	12 (3.3)	11 (12.4)	16 (7.8)	63 (7.3)
Missing/unknown	6 (2.9)	22 (6.2)	2 (2.2)	4 (1.9)	34 (3.9)

*At most recent birth.

†N=871 babies total (denominator).

Table 2 shows adolescent experiences of mistreatment during childbirth by country and age group. Experiences of any physical abuse, verbal abuse, or stigma and discrimination were high across countries: 44.3% (127/287) among 15–19 year olds, and 36.5% (210/575) among 20–24 year olds. In Ghana, Guinea and Nigeria, experiences of any physical or verbal abuse or discrimination were consistently higher among 20–24 year olds compared with 15–19 year olds; however in Myanmar, experiences of any physical or verbal abuse or discrimination was higher among 20–24 year olds. Across all countries, non-consented episiotomies were comparable across 15–19 year olds (n=20/287, 35.7%) and 20–24 year olds (n=47/575, 36.7%).

**Table 2:** Adolescent experiences of mistreatment during childbirth, by country

	Ghana, N=208		Guinea, N=359		Nigeria, N=89		Myanmar, N=206		Total, N=862	
	15–19 years (n=60)	20–24 years (n=148)	15–19 years (n=173)	20–24 years (n=186)	15–19 years (n=15)	20–24 years (n=74)	15–19 years (n=39)	20–24 years (n=167)	15–19 years (n=287)	20–24 years (n=575)
Any physical abuse, verbal abuse, or stigma and discrimination	29 (48.3%)	66 (44.6%)	82 (47.4%)	73 (39.2%)	9 (60%)	31 (41.9%)	7 (17.9%)	40 (24%)	127 (44.3%)	210 (36.5%)
Physical abuse	8 (13.3%)	12 (8.1%)	46 (26.6%)	33 (17.7%)	5 (33.3%)	12 (16.2%)	1 (2.6%)	9 (5.4%)	60 (20.9%)*	66 (11.4%)*
Verbal abuse	25 (41.7%)	61 (41.2%)	64 (37.0%)	58 (31.2%)	8 (53.3%)	29 (39.2%)	7 (17.9%)	33 (19.8%)	104 (36.2%)	181 (31.5%)
Stigma or discrimination	10 (16.7%)	7 (4.7%)	3 (1.7%)	5 (2.7%)	3 (20.0%)	5 (6.8%)	1 (2.6%)	3 (1.8%)	17 (5.9%)	20 (3.5%)
Informed consent										
No of caesarean section	8 (13.3%)	16 (10.8%)	19 (11%)	24 (12.9%)	0 (0%)	6 (8.1%)	10 (25.6%)	48 (28.7%)	37 (12.9%)	94 (16.3%)
Non-consented caesarean section	1 (12.5%)	3 (18.8%)	1 (5.3%)	2 (8.3%)	0 (0%)	0 (0%)	1 (10%)	4 (8.3%)	3 (8.1%)	9 (9.6%)
No of vaginal births	52 (86.7%)	132 (89.2%)	153 (88.4%)	162 (87.1%)	15 (100%)	68 (91.9%)	29 (74.4%)	119 (71.3%)	250 (87.1%)	480 (83.5%)
No of episiotomies†	14 (26.9%)	26 (19.7%)	28 (18.3%)	16 (9.9%)	5 (31.3%)	38 (56.7%)	9 (31.0%)	48 (40.3%)	56 (22.4%)	128 (26.7%)
Non-consented episiotomy	3 (21.4%)	10 (38.5%)	9 (32.1%)	7 (43.8%)	1 (20.0%)	9 (23.7%)	7 (77.8%)	21 (43.8%)	20 (35.7%)	47 (36.7%)
Vaginal examination (VE)										
No of VEs (among n=802 women with VE)	57 (95%)	144 (97.3%)	161 (93.1%)	176 (94.6%)	14 (93.3%)	71 (95.7%)	36 (92.3%)	143 (85.6%)	268 (93.4%)	534 (92.9%)
Non-consented VEs	14 (24.6%)	34 (23.6%)	54 (33.5%)	50 (28.4%)	9 (64.2%)	26 (36.6%)	9 (25%)	45 (31.4%)	86 (32.1%)	155 (29.0%)
Poor communication										
Health worker or staff did not listen or respond to her concerns	30 (50%)	44 (29.7%)	39 (22.5%)	39 (21%)	4 (26.7%)	12 (16.2%)	19 (48.7%)	76 (45.5%)	92 (32.1%)	171 (29.7%)
Abandonment/neglect										
Staff member not present when baby came out†	2 (3.8%)	3 (2.3%)	1 (0.7%)	0 (0%)	1 (6.3%)	2 (3%)	1 (3.4%)	1 (0.8%)	5 (1.7%)	6 (1.3%)
Woman waited for long period before attendance by health worker	9 (15%)	23 (15.5%)	16 (9.2%)	15 (8.1%)	2 (13.3%)	7 (9.5%)	5 (12.8%)	36 (21.6%)	32 (11.1%)	81 (14.1%)
Woman felt ignored, neglected or presence was a nuisance	10 (16.7%)	19 (12.8%)	14 (8.1%)	17 (9.1%)	1 (6.7%)	5 (6.8%)	4 (10.3%)	24 (14.4%)	29 (10.1%)	65 (11.3%)
Pain relief										
Woman requested pain relief but did not receive	9 (15%)	17 (11.5%)	64 (37.0%)	75 (40.3%)	1 (6.7%)	7 (9.5%)	12 (30.8%)	41 (24.6%)	86 (30.0%)	140 (24.3%)
Supportive care										
Not allowed to have a companion during labour and birth	20 (33.3%)	46 (31.1%)	92 (53.2%)	94 (50.5%)	9 (60.0%)	55 (74.3%)	1 (2.6%)	0 (0%)	122 (42.5%)*	195 (33.9%)*
Did not have a companion present at any point	37 (61.7%)	86 (58.1%)	22 (12.7%)	22 (11.8%)	9 (60.0%)	45 (60.8%)	0 (0%)	1 (0.6%)	68 (23.7%)	154 (26.8%)
Autonomy										
No access to oral fluids†	4 (7.7%)	19 (14.4%)	13 (8.5%)	28 (17.3%)	5 (31.3%)	30 (44.8%)	5 (17.2%)	14 (11.8%)	27 (10.8%)*	91 (19%)*
Women not told to eat†	23 (44.2%)	55 (41.7%)	18 (11.8%)	26 (16%)	11 (68.8%)	43 (64.2%)	0 (0%)	0 (0%)	52 (20.8%)*	124 (25.8%)*
Woman not told to and did not mobilise†	46 (54.1%)	110 (74.3%)	8 (4.6%)	17 (7%)	15 (93.3%)	63 (79.7%)	16 (48.7%)	63 (37.1%)	85 (28.6%)*	253 (42.4%)*
Woman not asked preferred birth position†	59 (98.3%)	145 (98%)	148 (85.5%)	147 (79.0%)	15 (100%)	73 (98.6%)	33 (84.6%)	142 (85.0%)	255 (88.9%)	507 (88.2%)
Woman/baby detained for not being able to pay hospital bill	10 (16.7%)	4 (2.7%)	11 (6.4%)	19 (10.2%)	1 (6.7%)	6 (8.1%)	4 (10.3%)	8 (4.8%)	26 (9.1%)	37 (6.4%)
Health systems										
Woman instructed to clean up own blood, urine, feces or amniotic fluid after birth	0 (0%)	3 (2%)	1 (0.6%)	3 (1.6%)	0 (0%)	1 (1.4%)	5 (12.8%)	28 (16.8%)	6 (2.1%)*	35 (6.1%)*
Staff asked woman or companion for bribe or gift	7 (11.7%)	19 (12.8%)	84 (48.6%)	100 (53.7%)	4 (26.7%)	9 (12.2%)	18 (46.2%)	74 (44.3%)	113 (39.4%)*	202 (35.1%)*
Curtains or other privacy measures not available	58 (96.7%)	137 (92.6%)	88 (50.9%)	107 (78.7%)	13 (86.7%)	61 (82.4%)	21 (53.8%)	90 (53.9%)	180 (62.7%)	395 (68.7%)

*χ^2^ test showed differences in the age groups, p<0.05.

†Among n=730 women with vaginal birth.

Among 802 (93.0%) of adolescents and young women who experienced at least one vaginal examination, approximately one-third reported experiencing a vaginal examination conducted without their consent (15–19 years: n=86/268, 32.1%; 20–24 years: n=155/534, 29.0%), which is consistent across countries and age groups. Among 730 adolescents with a vaginal birth, 11 (1.5%) gave birth without a staff member present. Some (63/862, 7.3%) adolescents or their babies were detained in the hospital for not being able to pay bills (15–19 years: n=26/287, 9.1%; 20–24 years: n=37/575, 6.4%), and younger adolescents (15–19 years) in Ghana were particularly at risk (n=10/60, 16.7%). Most adolescents did not have curtains, partitions or other privacy measures used (15–19 years: n=180/287, 62.7%; 20–24 years: n=395/575, 68.7%), particularly in Ghana (15–19 years: n=58/60, 96.7%; 20–24 years: n=137/148, 92.6%), Guinea (15–19 years: n=88/173, 50.9%; 20–24 years: n=107/186, 78.7%) and Nigeria (15–19 years: n=13/15, 86.7%; 20–24 years: n=61/74, 82.4%).

Table 3 shows the relationship between adolescents and young women’s experiences of mistreatment and their overall satisfaction with the care they received, measured by their rating of overall satisfaction with their birth experience and whether they would recommend the health facility to others. Among the adolescents who experienced physical abuse, 12.8% (98/767) were satisfied and 13.7% (105/768) would recommend the facility to others. For those who experienced verbal abuse, 28.8% (221/767) were satisfied and 29.3% (225/768) would recommend the facility to others. Only 16% of those who waited for long periods of time were satisfied (125/767) and would recommend the facility to others (126/768). Those who had curtains used for privacy were more satisfied (416/762, 54.6%) and would recommend the facility to others (416/762, 54.5%). About half of adolescents and young women who did not give consent for vaginal examinations were not satisfied with the care they received (355/708, 50.1%) or would not recommend the health facility to others (361/705, 51.2%). Similarly, just under half of adolescents and young women who were not told to mobilise (eg, to walk around or maintain upright positions during labour) were not satisfied with the care they received (293/652, 44.9%) or would not recommend the health facility to others (298/653, 45.6%).

**Table 3:** Associations of adolescent and young women’s experiences of mistreatment and satisfaction with care

Typology of mistreatment	Overall satisfaction with services received* (N=767)†		Recommend the facility to others‡ (N=768)†	
	n (%)	AOR (95% CI)§‡‡	n (%)	AOR (95% CI)§‡‡
Physical abuse				
No	669 (87.2)	Ref	663 (86.3)	Ref
Yes	98 (12.8)	0.32 (0.19 to 0.59)¶	105 (13.7)	0.48 (0.27 to 0.84)¶
Verbal abuse				
No	546 (71.2)	Ref	543 (70.7)	Ref
Yes	221 (28.8)	0.19 (0.12 to 0.31)¶	225 (29.3)	0.24 (0.15 to 0.38)¶
Consent before vaginal examination** (n=708 for satisfaction, n=705 for recommend)				
No	355 (50.1)	0.53 (0.33 to 0.60)¶	361 (51.2)	0.81 (0.52 to 1.3)
Yes	353 (49.9)	Ref	344 (48.8)	Ref
Long wait times				
Yes	125 (16.3)	0.23 (0.14 to 0.38)¶	126 (16.4)	0.28 (0.17 to 0.45)¶
No	641 (83.6)	Ref	641 (83.6)	Ref
Being told to mobilise or mobilised during labour†† (n=652 for satisfaction, n=653 for recommend)				
No	293 (44.9)	0.42 (0.20 to 0.85)¶	298 (45.6)	0.74 (0.38 to 1.5)
Yes	356 (54.6)	Ref	352 (53.9)	Ref
Curtains, partitions or other privacy measures used (n=762 for satisfaction; n=764 for recommend)				
No	346 (45.4)	0.85 (0.51 to 1.4)	348 (45.5)	0.73 (0.43 to 1.2)
Yes	416 (54.6)	Ref	416 (54.5)	Ref

*Denotes the percentage of women who were satisfied (strongly agree and agree) with their childbirth experiences.

†Where denominators differ from overall satisfaction (n=767) and recommend to others (n=768) due to unknown responses, this is indicated.

‡Denotes the percentage of women who would recommend (strongly agree and agree) the facility to others.

§ORs adjusted for marital status, education, previous births and country.

¶Significant at p<0.05.

**Consent defined as being informed and obtaining permission before vaginal examination.

††Among women with vaginal birth.

‡‡OR is not adjusted for age due to the homogeneity of sample of adolescent and young population.

AOR, adjusted OR.

Multivariable logistic regression models were used to evaluate the association between adolescents and young women’s experiences of mistreatment during childbirth, and their overall satisfaction with the services they received, and whether they were likely to recommend the facility. Adolescents and young women who were physically abused were 68% less likely to report being satisfied with care (AOR: 0.32, 95% CI: 0.19 to 0.59) and 52% less likely to recommend the health facility to others (AOR: 0.48, 95% CI: 0.27 to 0.84). Those who were verbally abused were 81% less likely to report being satisfied with care (AOR: 0.19, 95% CI: 0.12 to 0.31) and 76% less likely to recommend the health facility to others (AOR: 0.24, 95% CI: 0.15 to 0.38). Adolescents and young women who reported providing informed consent for vaginal examinations were 47% less likely to report being satisfied with care received (AOR: 0.53, 95% CI: 0.33 to 0.60); there was no relationship to whether she would recommend the health facility to others (AOR: 0.81, 95% CI: 0.52 to 1.3). Adolescents and young women who experienced long waiting times before being attended to by health workers were 77% less likely to report being satisfied with care (AOR: 0.23, 95% CI: 0.14 to 0.38) and 72% less likely to recommend the health facility to others (AOR: 0.28, 95% CI: 0.17 to 0.45), compared with women who reported shorter waiting times. Adolescents and young women who were told not to mobilise (walking and upright positions during early labour) or did not mobilise during labour were 58% less likely to report being satisfied with care received (AOR: 0.42, 95% CI: 0.20 to 0.85); there was no relationship to whether she would recommend the health facility to others (AOR: 0.74, 95% CI: 0.38 to 1.5).

As shown in online supplemental appendix 2, adolescents and young women with less education were more likely to report satisfaction with care received (p=0.004) and those who initiated breast feeding within 1 hour were more likely to be satisfied with care received (p=0.016).

10.1136/bmjgh-2021-007954.supp2 Supplementary data

## Discussion

We explored adolescent experiences of mistreatment during childbirth, their satisfaction with care and factors contributing to these experiences. Our analysis shows that adolescents and young women experience high levels of mistreatment, particularly verbal abuse. We found that adolescents and young women who were physically or verbally abused and did not provide consent for vaginal examinations were much less likely to report being satisfied with the care they received and less likely to recommend the health facility to others.

In comparing this study sample to the population of all women of reproductive age in our larger study,^16^ our new analysis shows that adolescents and young women consistently experience higher levels of physical abuse, verbal abuse, stigma and discrimination, non-consented care and poor communication. For instance, over 20% of 15–19 year olds experienced physical abuse, compared with 11% of all women.^16^ Over 44% of 15–19 year olds experienced any physical or verbal abuse or discrimination, compared with 35% of all women.^16^ Adolescents and young women who experienced physical or verbal abuse were less likely to report satisfaction with care or to recommend the facility to others, which was similar to the relationship between mistreatment and satisfaction with care in the sample of all women.^29^ Our formative qualitative research conducted in the same countries posits some explanations for why adolescents and young women may be more likely to experience mistreatment.^17 21–25^ In Nigeria, Guinea and Ghana, both women and health workers believed that adolescents and young women may be considered ‘difficult’ or ‘disobedient’ when in labour, thus justifying poor treatment for failing to listen to the health workers. Moreover, adolescents and young women shared their experiences of judgemental comments from health workers about their sexuality or sexual history at what was perceived to be a young age or while being unmarried. Health workers perceived labour pains as a consequence of ‘enjoying sexual activity’ and thus expected adolescents and young women to endure labour pains without complaint. While our qualitative research may propose some explanations for why adolescents and younger women experience more mistreatment compared with older women, these explanations should not be considered justification for mistreatment.

Moreover, recent research on adolescent sexual and reproductive health in Sub-Saharan Africa has illuminated the importance of understanding and responding to the intersecting identities, experiences and social conditions that influence disadvantage.^30–33^ Chandra-Mouli *et al* (2021) argue that intersectionality has largely been ignored in the context of adolescent sexual and reproductive health, thus representing a critical gap in ‘holistic understanding of the way in which social systems, power and identity’ interact in adolescents’ lives and health.^30^ In our study, we were able to explore some of these social conditions (age, education, marital status, obstetric history), we did not have sufficient power to explore how other social conditions (such as class, religion, ethnicity or race, dis(ability) status and age at marriage) might contribute to better or worse experiences for adolescents during childbirth. More work is critically needed to further explore what groups of adolescents may experience greater risks of mistreatment during childbirth—and their poorer experiences of sexual and reproductive healthcare broadly speaking—to generate actionable evidence to address these inequalities and ensure healthcare services are accessible and acceptable to all.

In Ghana, over 96% of 15–19 year olds and 92% of 20–24 year olds did not have curtains or privacy measures used, compared with approximately 8% of all women.^16^ In this context, most women in the study hospitals were admitted in labour to an open labour ward without private space, although some facilities had private birthing suites. Sometimes curtains were used to provide privacy, but they were typically folded and not used optimally. This is an important finding that needs urgent attention and action. Adolescents and young women are a special group that require optimal privacy to improve their experience of care and satisfaction with the services they receive, particularly during childbirth which may be a time of additional anxiety and stress. We suggest that to cater for adolescents’ need for optimal privacy, partitioning of the birthing suites should be done and there should be adequate provision and effective use of curtains. Non-consented care was a major occurrence that needs further attention, especially obtaining informed consent prior to performing vaginal examinations and episiotomy. This is partly attributed to a lack of respect and autonomy for the adolescents and a lack of an adolescent-friendly environment.

Our paper presents one of the first analyses focused on adolescents and young women’s experiences of mistreatment during childbirth and shows clear relationships between younger age and poor care experiences. Key strengths of this study included the use of evidence-informed tools and facilitation of interviews by non-clinical female data collectors (to reduce social desirability bias and potential under-reporting of negative birth or care experiences). A limitation of our study design is that all study sites were secondary and tertiary facilities, which means that the experiences reported by adolescents and young women may not be generalisable to other settings. As with all surveys using Likert scales, our use of Likert scales made it challenging to distinguish between strongly and weakly held opinions, limiting the precision in the participants’ ability to meaningfully distinguish between the five response options. We aimed to mitigate these limitations by having a clear group that agreed (strongly agreed/agreed) with the questions on their satisfaction with the care that they received and their willingness to recommend their health facilities to others. Other responses that is strongly disagreed/disagreed/neutral were grouped together to reflect a range of results showing not optimal experiences. The study sample size was calculated for the study population aged 15–49 years, and this analysis represents 862 adolescents and young women, or approximately one-third of the original sample size. As such, these results may be considered as hypothesis generating and indicate that further work is needed to unpack the complex determinants influencing poorer treatment of adolescents and young women in maternity care globally. This includes more robust exploration of which groups of adolescents may be at higher risk of poor treatment based on their social position, such as socioeconomic status and class, religion, ethnicity or race, dis(ability) status and gender expression.

## Conclusion

Adolescent morbidity and mortality from pregnancy and childbirth complications remain persistent global health problems. The high mistreatment and low satisfaction with childbirth in health facilities among adolescents and young women, as shown in this study, suggest that these young women are high-risk groups that consistently receive poorer care compared with older women. Critical action is needed to ensure that existing maternity health services are responsive to the unique needs of adolescents and young women. This may include paying particular attention to their needs and ensuring that adolescents and young women have access to interventions that improve social and emotional support during labour and are potentially acceptable to youth, such as labour companionship.^34^

The most common form of mistreatment among adolescents and young women is verbal abuse which cuts across all age groups and across all countries. Other forms of mistreatment experienced by adolescents across countries were very high levels of poor communication, lack of supportive care and lack of privacy. Experiences of mistreatment greatly affects adolescents’ and young women’s satisfaction with the care they received, and more work is urgently needed to provide services that are more adolescent-friendly and non-judgmental. While adolescents and young women should be supported to use their own agency in maternal healthcare seeking, it is critical to recognise their unique needs and how their needs may intersect with social stigma around sex and pregnancy. More work is needed to ensure that adolescents and young women’s needs and preferences are met in maternity care settings globally, particularly around improving equitable and non-discriminatory care.

## Data Availability

Data are available on reasonable request.

## References

[1] Chandra-Mouli V , Ferguson BJ , Plesons M , et al . The political, research, programmatic, and social responses to adolescent sexual and reproductive health and rights in the 25 years since the International Conference on population and development. J Adolesc Health 2019;65:S16–40. 10.1016/j.jadohealth.2019.09.011 31761001

[2] World Health Organization . Global accelerated action for the health of adolescents (AA-HA!): guidance to support country implementation.

[3] UN DESA Population Division . Adolescent fertility since the International Conference on population and development (ICPD) in Cairo. New York, 2013.

[4] UNFPA . Girlhood, not motherhood: preventing adolescent pregnancy. New York, 2015.

[5] World Health Organization . Global and regional estimates on violence against women: prevalence and health effects of intimate partner violence and non-partner sexual violence. Geneva, Switzerland: WHO, 2013.

[6] Kozuki N , Lee ACC , Silveira MF , et al . The associations of birth intervals with small-for-gestational-age, preterm, and neonatal and infant mortality: a meta-analysis. BMC Public Health 2013;13:S3. 10.1186/1471-2458-13-S3-S3 PMC3847557 24564484

[7] UNICEF . Ending child marriage: progress and prospects. New York: UNICEF, 2013.

[8] UNFPA . Adolescent and youth dashboard. New York: UNFPA, 2021.

[9] Ganchimeg T , Ota E , Morisaki N , et al . Pregnancy and childbirth outcomes among adolescent mothers: a World Health Organization multicountry study. BJOG 2014;121:40–8. 10.1111/1471-0528.12630 24641534

[10] UNFPA . State of the world midwifery 2021. New York: UNFPA, 2021.

[11] World Health Organization . Global standards for quality health care services for adolescents.

[12] Raj A , Boehmer U . Girl child marriage and its association with national rates of HIV, maternal health, and infant mortality across 97 countries. Violence Against Women 2013;19:536–51. 10.1177/1077801213487747 23698937

[13] Bohren MA , Vogel JP , Hunter EC , et al . The mistreatment of women during childbirth in health facilities globally: a mixed-methods systematic review. PLoS Med 2015;12:e1001847. 10.1371/journal.pmed.1001847 26126110 PMC4488322

[14] World Health O . WHO statement: the prevention and elimination of disrespect and abuse during facility-based childbirth. Geneva, Switzerland, 2014.

[15] World Health O . WHO recommendations: intrapartum care for a postive childbirth experience. Geneva, Switzerland: World Health Organization, 2018. 30070803

[16] Bohren MA , Mehrtash H , Fawole B , et al . How women are treated during facility-based childbirth in four countries: a cross-sectional study with labour observations and community-based surveys. Lancet 2019;394:1750–63. 10.1016/S0140-6736(19)31992-0 31604660 PMC6853169

[17] Maya ET , Adu-Bonsaffoh K , Dako-Gyeke P , et al . Women’s perspectives of mistreatment during childbirth at health facilities in Ghana: findings from a qualitative study. Reprod Health Matters 2018;26:70–87. 10.1080/09688080.2018.1502020 30152268

[18] Warren CE , Njue R , Ndwiga C , et al . Manifestations and drivers of mistreatment of women during childbirth in Kenya: implications for measurement and developing interventions. BMC Pregnancy Childbirth 2017;17:102. 10.1186/s12884-017-1288-6 28351350 PMC5371243

[19] Amroussia N , Hernandez A , Vives-Cases C , et al . “Is the doctor God to punish me?!” An intersectional examination of disrespectful and abusive care during childbirth against single mothers in Tunisia. Reprod Health 2017;14:32. 10.1186/s12978-017-0290-9 28259180 PMC5336668

[20] Shakibazadeh E , Namadian M , Bohren MA , et al . Respectful care during childbirth in health facilities globally: a qualitative evidence synthesis. BJOG 2018;125:932–42. 10.1111/1471-0528.15015 29117644 PMC6033006

[21] Balde MD , Bangoura A , Diallo BA , et al . A qualitative study of women’s and health providers' attitudes and acceptability of mistreatment during childbirth in health facilities in guinea. Reprod Health 2017;14:4. 10.1186/s12978-016-0262-5 28086975 PMC5237275

[22] Balde MD , Diallo BA , Bangoura A , et al . Perceptions and experiences of the mistreatment of women during childbirth in health facilities in guinea: a qualitative study with women and service providers. Reprod Health 2017;14:3. 10.1186/s12978-016-0266-1 28077145 PMC5225581

[23] Bohren MA , Vogel JP , Tunçalp Özge , et al . Mistreatment of women during childbirth in Abuja, Nigeria: a qualitative study on perceptions and experiences of women and healthcare providers. Reprod Health 2017;14:9. 10.1186/s12978-016-0265-2 28095911 PMC5240205

[24] Bohren MA , Vogel JP , Tunçalp Ö , et al . "By slapping their laps, the patient will know that you truly care for her": A qualitative study on social norms and acceptability of the mistreatment of women during childbirth in Abuja, Nigeria. SSM Popul Health 2016;2:640–55. 10.1016/j.ssmph.2016.07.003 28345016 PMC5356417

[25] Maung TM , Show KL , Mon NO , et al . A qualitative study on acceptability of the mistreatment of women during childbirth in Myanmar. Reprod Health 2020;17:56. 10.1186/s12978-020-0907-2 32312305 PMC7171855

[26] Vogel JP , Bohren MA , Tunçalp Özge , et al . How women are treated during facility-based childbirth: development and validation of measurement tools in four countries - phase 1 formative research study protocol. Reprod Health 2015;12:60. 10.1186/s12978-015-0047-2 26198988 PMC4510886

[27] Bohren MA , Vogel JP , Fawole B , et al . Methodological development of tools to measure how women are treated during facility-based childbirth in four countries: labor observation and community survey. BMC Med Res Methodol 2018;18:132. 10.1186/s12874-018-0603-x 30442102 PMC6238369

[28] Scott K , Gharai D , Sharma M , et al . Yes, no, maybe so: the importance of cognitive interviewing to enhance structured surveys on respectful maternity care in northern India. Health Policy Plan 2020;35:67–77. 10.1093/heapol/czz141 31670773 PMC7053388

[29] Maung TM , Mon NO , Mehrtash H , et al . Women’s experiences of mistreatment during childbirth and their satisfaction with care: findings from a multicountry community-based study in four countries. BMJ Glob Health 2021;5:e003688. 10.1136/bmjgh-2020-003688 PMC7816916 33436494

[30] Chandra-Mouli V , Neal S , Moller A-B . Adolescent sexual and reproductive health for all in sub-Saharan Africa: a spotlight on inequalities. Reprod Health 2021;18:118. 10.1186/s12978-021-01145-4 34134737 PMC8208826

[31] Melesse DY , Cane RM , Mangombe A , et al . Inequalities in early marriage, childbearing and sexual debut among adolescents in sub-Saharan Africa. Reprod Health 2021;18:117. 10.1186/s12978-021-01125-8 34134718 PMC8210338

[32] Wado YD , Mutua MK , Mohiddin A , et al . Intimate partner violence against adolescents and young women in sub-Saharan Africa: who is most vulnerable? Reprod Health 2021;18:119. 10.1186/s12978-021-01077-z 34134704 PMC8210343

[33] Mutua MK , Wado YD , Malata M , et al . Wealth-related inequalities in demand for family planning satisfied among married and unmarried adolescent girls and young women in sub-Saharan Africa. Reprod Health 2021;18:116. 10.1186/s12978-021-01076-0 34134700 PMC8210345

[34] Bohren MA , Hofmeyr GJ , Sakala C , et al . Continuous support for women during childbirth. Cochrane Database Syst Rev 2017;2017:CD003766. 10.1002/14651858.CD003766.pub6 PMC6483123 28681500

