# Supplementary material for: Adolescent experiences of mistreatment during childbirth in health facilities: secondary analysis of a community-based survey in four countries
Source: BMJ Glob Health. 2022 Mar 21;5(Suppl 2):e007954. doi: 10.1136/bmjgh-2021-007954 (PMC10175942; doi:10.1136/bmjgh-2021-007954)
Supplement: Supplementary data [file bmjgh-2021-007954supp001.pdf]

# Appendix S1 – Reflexivity Statement

**1. How does this study address local research and policy priorities?**

The overall study was designed as a research collaboration to address the pressing issue of the mistreatment of women during childbirth, which was identified in the study countries (Nigeria, Ghana, Guinea, Myanmar) as a pressing issue affecting maternal health. This study started as a 3-country study (Nigeria, Ghana and Myanmar) and the team from Guinea (CERREGUI) joined our collaboration several months into the planning stage, after they submitted a similar project to the Human Reproduction Programme (HRP) Alliance for Research Capacity Strengthening as part of their long-term institutional development programme plan. For this particular analysis and paper, the team from Nigeria (TAI, AKA, OIF, OA) developed the research questions and analysis plan based on their local priorities related to high rates of adolescent pregnancy, early marriage, early sexual initiation, and corresponding mistreatment during birth.

**2. How were local researchers involved in study design?**

The overall study design was co-developed through consultation with the whole study group. At an initial project meeting at WHO in 2014, the country research teams (KAB – Ghana, TMM – Myanmar, MDB – Guinea) brainstormed appropriate study designs based on their context and in consultation with the WHO research team (OT, JPV, MAB). We note with sadness that Prof Bukola Fawole (Nigeria principal investigator) passed away during the project, but participated in these design meetings and led the Nigeria team throughout data collection. TAI, AKA, and OA were study site coordinators in Nigeria and subsequently supported analysis and writing of several papers on behalf of the Nigeria team. OIF is a specialist on violence against women and adolescent health in Nigeria, as well as the wife of the late Prof Fawole and was instrumental in contributing her expertise to this study. TAI and AKA led the analysis with support from HM, OT and MAB. MP and VCM joined the research team for this analysis based on their global expertise on adolescent health and particularly to contribute to framing the research questions and interpreting the research and policy implications of the findings.

**3. How has funding been used to support the local research team?**

At the start of the overall study, we developed a research capacity strengthening plan with the local research teams and WHO researchers, with support from the HRP Alliance for Research Capacity Strengthening. During the project, WHO research team (MAB, JPV, OT) co-facilitated two week-long workshops on data analysis (1-Accra, Ghana 2015, with >20 members of the Nigeria, Ghana and Guinea research teams including KAB, MDB and others not named on this particular paper, 2-Yangon, Myanmar 2015, with 14 members of the Myanmar research team and other junior researchers from the Department of Medical Research) and three week-long workshops on scientific writing (1-Conakry, Guinea 2016, 2-Yangon, Myanmar 2016, 3-Melbourne, Australia 2019). Funding has also been used to facilitate post-study dissemination workshops in Guinea, where the team led by MDB shared the results and implications from the study with the Ministry of Health, professional associations, WHO-Guinea and other key stakeholders: https://www.who.int/news/item/15-05-2020-research-leads-to-actions-improving-childbirth-in-guinea. The overall study has also contributed to KAB and TMM's PhD dissertations (MAB co-supervised TMM's PhD through Khon Kaen University Thailand, where his PhD scholarship was supported by the HRP Alliance for Research Capacity Strengthening).

**4. How are research staff who conducted data collection acknowledged?**

The paper is one of 16 papers (7 qualitative from the formative phase, 1 protocol, 1 methodological development, and 7 quantitative from the measurement phase) from our research collaboration. Across these 16 papers, research staff responsible for data collection from all 4 countries have contributed as co-authors on at least 1 paper. Each country research team has led at least 2 papers (1 qualitative, 1 quantitative) with their teams. All other research staff contributing to data collection have been acknowledged.

**5. Do all members of the research partnership have access to study data?**

All members of the partnership have access to data and have led analysis of data in this and other papers (see #4).

**6. How was data used to develop analytical skills within the partnership?**

The research team worked collaboratively on data analysis across the whole study and in this particular paper. Two week-long data analysis workshops and three week-long scientific writing workshops were facilitated as part of the research partnership to strengthen the analytic and writing skills for the research team (see #3). For this paper, TAI and AKA led the analysis with support from HM, OT and MAB.

**7. How have research partners collaborated in interpreting study data?**

As mentioned in #3 and #6, all research partners have collaborated to interpret the study data during data analysis and scientific writing workshops held across the project. For each multi-country paper produced from our research partnership, discussions were facilitated to understand and co-develop the implications for research, policy and practice for each country.

**8. How were research partners supported to develop writing skills?**

The research team writing this paper is a mix of senior, mid, and junior academics and clinicians. OIF, TMM, JPV and MAB have completed their PhDs in the last 7 years, and KAB, AKA and HM are currently working on their PhDs. The authorship team was supported by the WHO research team (MAB, OT) to develop and refine writing skills, including through the facilitation of scientific writing workshops (see #3).

**9. How will research products be shared to address local needs?**

All papers resulting from our research partnership have been published as open access. We have developed a post-publication dissemination plan within WHO and our respective institutions to share our research widely across our networks. The study tools are available freely in eight languages for other research teams to access.

**10. How is the leadership, contribution and ownership of this work by LMIC researchers recognised within the authorship?**

Authors TAI and AKA led this work and are first, second and corresponding authors respectively. We note that the majority of authors are researchers from the study countries (Nigeria, Ghana, Guinea, Myanmar).

11. **How have early career researchers across the partnership been included within the authorship team?**

We have included early career researchers and clinician researchers (TAI, AKA, OIF, OA, HM, KAB, TMM, MAB) within the authorship team. They have contributed to the data collection, analysis plan, analysis, and writing. We acknowledge that 2 of 8 of the early career researchers are based in high-income countries, and the remaining 6 are based in the study countries (Nigeria, Ghana, Guinea, Myanmar).

12. **How has gender balance been addressed within the authorship?**

Six authors are female (TAI, OIF, HM, MP, OT, MAB) and seven are male (AKA, OA, KAB, TMM, MDB, JPV, VCM).

13. **How has the project contributed to training of LMIC researchers?**

The overall study has also contributed to KAB and TMM's PhD dissertations (MAB co-supervised TMM's PhD through Khon Kaen University Thailand, where his PhD scholarship was supported by the HRP Alliance for Research Capacity Strengthening). Please see #3 about analysis and writing workshops facilitated for this project, which were specifically designed by the research teams to address research training needs in each country.

14. **How has the project contributed to improvements in local infrastructure?**

This project has not directly contributed to improvements in local infrastructure.

15. **What safeguarding procedures were used to protect local study participants and researchers?**

Adaptations to the study were made based on the local contexts to safeguard our research team. For example, in Myanmar it was not appropriate or safe to conduct labour observations in the maternity ward of public hospitals, so this component of the project was not implemented in Myanmar. Regular debriefing and reflexive discussions between data collectors, country principal investigators and the WHO research team helped to address any issues arising during data collection – this process of reflection during data collection is critical to any research involving sensitive topics or violence to ensure the safeguarding of research participants and research teams.
