# Supplementary material for: Adolescent experiences of mistreatment during childbirth in health facilities: secondary analysis of a community-based survey in four countries
Source: BMJ Glob Health. 2022 Mar 21;5(Suppl 2):e007954. doi: 10.1136/bmjgh-2021-007954 (PMC10175942; doi:10.1136/bmjgh-2021-007954)
Supplement: Supplementary data [file bmjgh-2021-007954supp002.pdf]

Appendix S2: Socio-demographic and Obstetric factors potentially associated with the level of satisfaction of care among adolescents

	N (%)	Satisfied with care received (agree/strongly agree)	Not satisfied with care received (disagree/strongly disagree/neutral)	P value
**Age(years)**				
15-19	287(33.3%)	258(33.6%)	29(30.5%)	0.186
20-24	575(66.7%)	509(66.4%)	66(69.5%)	
**Marital Status**				
Married/Cohabiting	723(83.9%)	646(84.2%)	77(81.1%)	0.129
Single/separated/divorced	138(16.0%)	121(15.8%)	17(17.9%)	
Unknown	1(0.1%)	-	1(1.1%)	
**Educational Status of the women**				
No Education	125(14.5%)	111(14.5%)	14(14.7%)	0.004*
Some Primary education	118(13.7%)	101(13.2%)	17(17.9%)	
Some secondary education	288(33.4%)	260(33.9%)	28(29.5%)	
Complete secondary education	260(30.2%)	227(29.6%)	33(34.7%)	
Complete tertiary education	63(7.3%)	61(7.9%)	2(2.1%)	
Vocational/others	8(0.9%)	7(0.9%)	1(1.1%)	
**Previous number of times pregnant**				
1	539(62.5%)	480(62.6%)	59(62.1%)	0.459
2	209(24.3%)	187(24.4%)	22(23.2%)	
3	76(8.8%)	69(9%)	7(7.4%)	
≥4	37(4.3%)	30(3.9%)	7(7.4%)	
Unknown	1(0.1%)	1(0.1%)	-	
**Previous number of births**				
1	703(81.6%)	630(82.1%)	73(76.8%)	0.279
2	109(12.6%)	92(12.1%)	17(17.9%)	
3	37(4.3%)	34(4.4%)	3(3.2%)	
≥4	13(1.5%)	11(1.4%)	2(2.1%)	
**Mode of birth**				
Vaginal birth	730(84.7%)	652(85%)	78(82.1%)	0.334
Caesarean section	131(15.2%)	115(15%)	16(16.8%)	
Unknown	1(0.1%)	-	1(1.1%)	
**Number of babies**				
1	849(98.5%)	755(98.4%)	94(98.9%)	0.731
≥2	13(1.5%)	12(1.6%)	1(1.1%)	
**Sex of baby**				
Male	458(53.1%)	402(52.4%)	56(58.9%)	0.650
Female	402(46.6%)	364(47.5%)	38(40.0%)	
Unknown	2(0.3%)	1(0.1%)	1(1.1%)	
**Breastfeeding initiation time**				
Within 1 hour	404(46.9%)	373(48.6%)	31(32.6%)	0.016*
Within 24 hours	361(41.9%)	315(41.1%)	46(48.4%)	
Within 1 week	59(6.8%)	51(6.6%)	8(8.4%)	
Longer than 1 week	4(0.5%)	3(0.4%)	1(1.1%)	
Unknown/missing	34(3.9%)	25(3.3%)	9(9.5%)	

* significant level p<0.05
